# The complete mitochondrial genome of an endangered tree: *Malania oleifera*

**DOI:** 10.1080/23802359.2020.1841583

**Published:** 2020-12-24

**Authors:** Hang Luo, Jie Xu, Si-Qian Jiao, Ren-Gang Zhang, Jian-Feng Mao

**Affiliations:** a College of Biological Sciences and Technology, Beijing Advanced Innovation Center for Tree Breeding by Molecular Design, National Engineering Laboratory for Tree Breeding, Beijing Forestry University, Beijing, China; b Beijing Ori-Gene Science and Technology Co. Ltd, Beijing, China

**Keywords:** *Malania oleifera*, endangered species, mitochondrial genome

## Abstract

*Malania oleifera* is an endangered species found in restricted areas in Karst areas in Southwestern China and is also with significant economic and ecological values. Here, complete mitochondrial genome of *M. oleifera* was characterized, which is the first for the Olacaceae family. The mitogenome is 527,575 bp in length with a GC content of 45.65%, including one pseudogene, and 38 protein-coding, 32 tRNA, three rRNA genes. Eleven genes have two copies in the mitogenome, and 3 genes are trans-spliced. Phylogenetic tree found that *M. oleifera* is making a sister branch to that of species from Rosids and Asterids.

*Malania oleifera* Chun & SK Lee, an endemic evergreen tree belonging to the monotypic genus *Malania* of the Olacaceae family (Guo et al. 2018), is naturally distributed in restricted Karst region of southeast Yunnan and west Guangxi province of China (N22°23′–N24°28′ in latitude and E105°30′–E107°30′ in longitude) (Li et al. 2019). It can be used as an excellent ecological tree to control rocky desertification because its root system could be well-developed in that extreme habitat (Lv et al. 2016). Besides, it has high economic and medicinal value as its seed contains very high content of nervonic acid (55.7–67%) (Ma et al. 2004). *M. oleifera* has been listed in the IUCN Red List as B1 + 2c criteria of VU category (extent of occurrence estimated to be <20,000 km^2^ and a continuing decline, observed, projected, or inferred, in numbers of mature individuals) (Sun 1998) and assigned as a plant of extremely small population size need urgent conservation (Ma et al. 2013). In this study, we provided the full genome assembly of the mitogenome of *M. oleifera*, which represents the first complete mitogenome within the Olacaceae and second within the order Santalales. Availability of *M. oleifera* mitogenome provides basic information for conservation, evolutionary, and functional studies.

Fresh leaves from a mature healthy plant growing in Guangnan County, Yunnan Province (N23.90°, E104.90°) were collected and genome-wide DNA extraction was conducted. The voucher specimen (accession no. mol20170910) was deposited in the Herbarium of Beijing Forestry University (BJFC), Beijing, China. Then, PacBio SMRTbell sequencing libraries and GemCode sequencing library were built, respectively. PacBio SMRT sequencing was performed on a PacBio Sequel instrument using S/P2-C2 sequencing chemistry (10 SMRT cells). 5,778,035 PacBio long reads (51.15 Gb, roughly 30× coverage) were generated. The GemCode library was sequenced using 2 × 150 paired-end (PE) sequencing on Illumina HiSeq X Ten. In total, 899.778 million short reads (∼134.97 Gb, roughly 89× coverage) were obtained (Xu et al. 2019). High-quality reads were used for mitochondrial assembly. GetOrganelle v1.6.2e (https://github.com/Kinggerm/GetOrganelle) (Jin et al. 2019) was used to isolate mitochondria reads then assembled into graph combining with correction and simplification. Mitochondrial reads of both PacBio and Illumina sequencing were aligned to the assembled graph with Minimap2 v2.17 (r941) (Li 2018), and then the alignment was used to improve the accuracy of the assembled graph. Finally, a circular mitogenome was gained. OGAP pipeline (https://github.com/zhangrengang/OGAP) was further used to identify genes. Exonerate v2.2.0 (Slater and Birney 2005) and AUGUSTUS v3.3.1 (Stanke et al. 2006) were performed for annotated protein-coding genes. All tRNA genes were confirmed by tRNAscan-SE v2.0.5 (Lowe and Eddy 1997) and rRNA gene was verified by BLAT v36 (Kent 2002).

The complete mitochondrial genome sequence of *M. oleifera* (GenBank accession number MT902145) is 527,575 bp in length, and base composition is A (27.14%), T (27.22%), C (22.76%), and G (22.89%). This mitogenome contains one pseudogene (rps13) and 38 protein-coding genes that are involved in NADH dehydrogenase (nad1, 2, 3, 4, 4L, 5, 6, 7, 9), cytochrome c oxidase (cox1, 2, 3), cytochrome c biogenesis (ccmB, C, FN, FC), apocytochrome b (cob), ATP synthase (atp1, 4, 6, 8, 9), ribosomal proteins (rpl2, 5, 10, 16 and rps 1, 3, 4, 7, 10, 12, 14,19), succinate dehydrogenase subunits (sdh3, sdh4), maturase (matR), and MttB gene. Among these genes, 11 genes (atp4, ccmC, ccmFC, cob, cox2, cox3, nad4L, rpl5, rps7, rps12, rps14) have two copies, and three genes (nad1, nad2, nad5) were trans-spliced. Moreover, this mitogenome contains 32 tRNA genes coding for 16 amino acid and three rRNA genes (rrn5, 18, 26).

For phylogenetic analysis, complete mitochondrial genomes from other eight species were selected. Protein-coding genes were aligned using MAFFT v7.221 (Katoh et al. 2002). The maximum likelihood phylogenetic tree was constructed by IQ-TREE v1.6.5 (Nguyen et al. 2015) with the model of GTR + F + R2 and 1000 bootstrap replicates. *M. oleifera* is making an isolated branch and a sister to the branch making by species from Rosids (*Arabidopsis thaliana*, *Gossypium raimondii*, and *Salix suchowensis*) and Asterids (*Vaccinium macrocarpon*, *Solanum lycopersicum*, and *Helianthus annuus*) (Figure 1).

**Figure 1.**
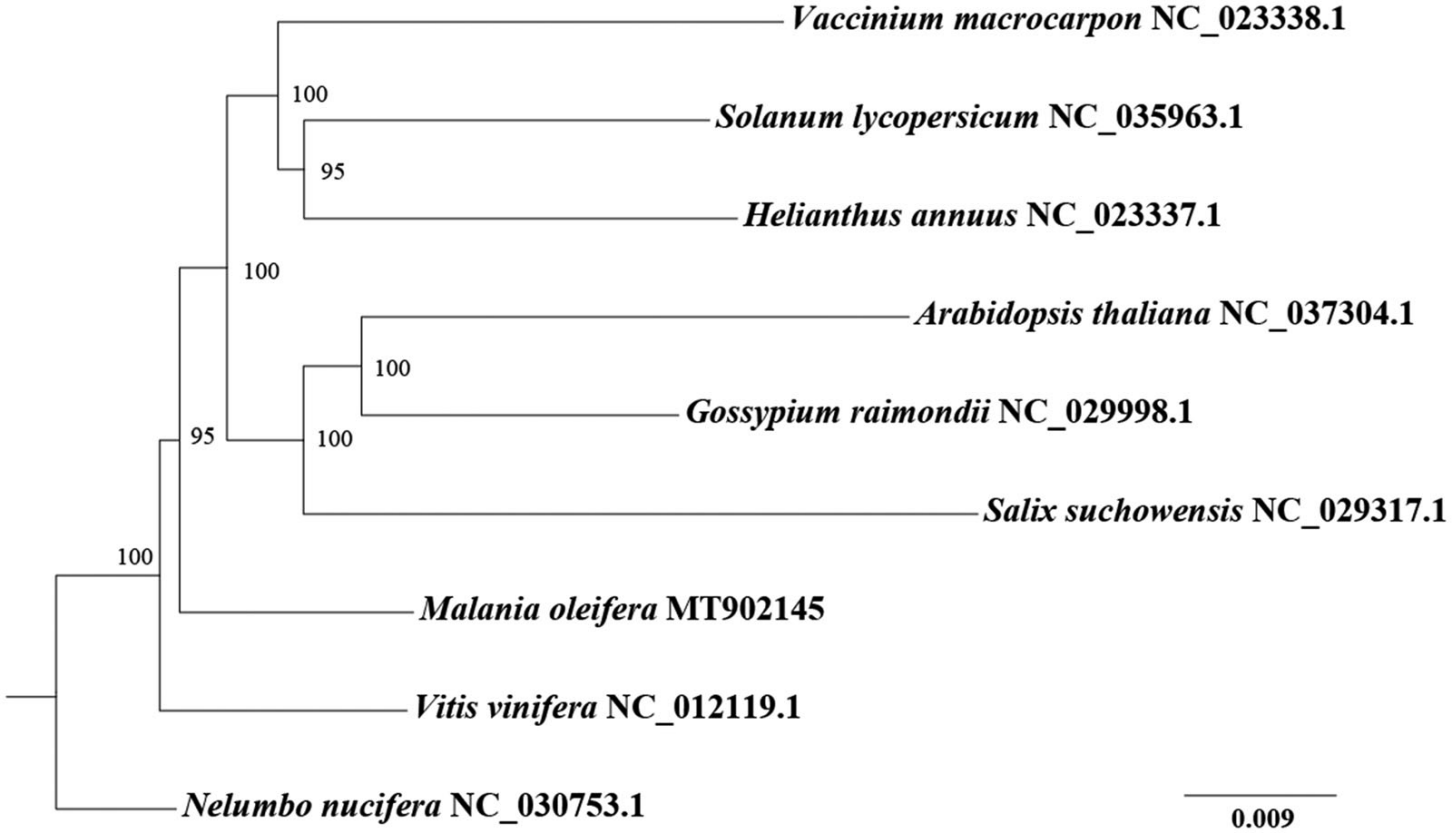
The maximum likelihood tree based on protein-coding genes of *M. oleifera* and other eight species. The numbers on the nodes indicate bootstrap values from 1000 replicates.

## Data Availability

The data that support the findings of this study are openly available in GenBank (https://www.ncbi.nlm.nih.gov/) under reference number MT902145.
